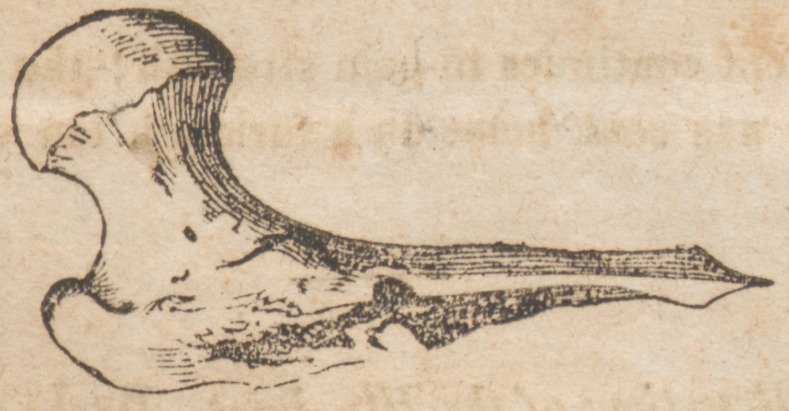# Resections of the Hip-Joint

**Published:** 1864-01

**Authors:** James B. Read

**Affiliations:** Surg. P. A. C. S.


ART. II.—*Resections of the Hip-Joint.* By JAMES B. READ, *Surg. P. A. C. S.*

James M. Jarrett, lieutenant company "C," 15th N. C., a native of North Carolina; 28 years of age; fair complexion; medium size and spare habit; general health good; of temperate habits; occupation, before the war, an upholsterer; entered general hospital No. 4, Richmond, Va., October 20th, 1863; at the affair at Bristow, October 14th, was wounded by a Minie ball, entering in front and rather to the outside of the median line of the left thigh, about two inches below Poupart's ligament, fracturing and comminuting the femur, and making its exit on the outer and lower side of the limb, rather higher than the point of entrance. A straight splint had been applied to the limb, and he had been transported one hundred and sixty miles over a rough country road in an ambulance. He was much exhausted when received in the hospital, and complained of great pain on any movement

Bandages and splints were at once removed, and the thigh placed comfortably on pillows. The limb was swollen and red, both orifices discharging bloody pus. Chloroform had to be administered before he would permit the dressing to be changed. Wet dressings were applied to the thigh and an opiate administered.

The case from this time progressed unfavorably. He gradually lost flesh; the pulse became quick and small; the tongue dry and red; and there was a tendency to run off by the bowels.

The discharges from the wounds purulent and bloody, mixed with bubbles of offensive gas; occasionally small pieces of bone came away; night sweats were of frequent occurrence.

Nov. 9th.—Jarrett's condition growing gradually worse, a consultation was asked for, and surgeons C. B. Gibson and M. Michel saw the patient with me. After a careful examination of the case, it was determined that it was one that called for operative interference as the best chance for recovery. The patient was cheerful, and desired to be relieved from the agony which he endured on the slightest movement.

4 P. M.—Chloroform having been previously administered, being placed on the table on his right side, a straight incision was made about two inches below the point of exit, and carried through this over the trochanter major two inches' making a wound of about seven inches in length; the lower end of the femur was now examined, and was found pointed and jagged, with a thin plane of bone split off of its anterior surface for about three inches.

This portion of the femur was protruded from the wound by an assistant pushing it up and carrying the leg over the other thigh; the soft parts were protected by a wooden spatula, and two inches were sawed off. The upper portion was then sought for and the sharp end of the upper fragment of the femur was found drawn directly across the thigh by the contraction of the psoas magnus and the illiacus internus muscles. After several ineffectual efforts to dislodge this from its position, it was seized with the lesion forceps, and by the exertion of great force, the contraction of the muscles was sufficiently overcome to permit of their attachment to the trochanter minor being divided. The capsule of the joint was now opened. It was found impossible, owing to the shortness of this portion of the bone left, to dislocate with facility the head of the femur, so as to prevent the division of the ligamentum teres. Partial dislocation was produced by twisting the head of the bone violently at the ligament divided.

The wound was now cleansed of many large fragments and sharp spiculæ of bone that were imbedded in clots, from which they had to be enucleated with the finger nail.

The wound was closed with sutres and adhesive strips; dry dressings were used; a long splint was loosely applied, so as not to prevent muscular contraction, but to keep the thigh fixed.

Two grains of opium and one and a quarter drachm brandy were directed to be given every two hours.

10 P. M. Pulse 120; patient comfortable, and expressed himself as much relieved from pain, and able to move with ease and comfort.

There have been no unpleasant symptoms since the operation; the wound did remarkably well; showed a disposition to early union.

The patient has gained strength; his appetite and digestion are good at this time (Dec 9th); his condition is as follows: Pulse 76, full and natural; tongue healthy; appetite good; sleeps well at night; is cheerful, and complains of no pain. The limb is shortened five inches; the wound has healed almost entirely.

There is a slight discharge from two points, one leading to the cotyloid cavity and the other to the bone. The cicatrix has contracted, and there seems to be a disposition to contract round the end of the bone. The amount of pus discharged is not a half an ounce in twenty-four hours.

The muscles feel firm and natural; he can move himself with facility and ease in his bed.

*Case 2.*

Alfred Toney, private 16th N. C.; wounded June 30th, 1863; entered hospital same day, with, apparently, a wound of left buttock, the ball remaining. No particular attention was called to this case for some time. He seemed to be doing well.

August 11th. He complained of great pain in the knee and ankle; the slightest touch produced great anguish; considerable œdema of the foot existed. Chloroform was administered, and the wound examined. The finger could not pass deeper than half an inch in the wound until the limb was carried forward; then it passed to the cotyloid cavity; the ball was felt in the acetabalum; the round ligament was severed and the head of the bone slightly fractured, and deprived of all of its cartilage. The patient was laid on his face by the edge of the table; an incision on the trochanter major, down through the tissues to the joint, was made, as in the last case.

The head of the femur was dislocated by forcibly bringing the leg under the table, a spatula placed to protect the tissues, and the head of the bone was sawed off. The bullet was then removed from the cavity.

The cotyloid cavity was then found to be broken across and the cartilage loosened.

The wound was closed with sutures, and the patient removed to bed.

Patient was more comfortable, had no pain, and in twenty-four hours the swelling had entirely subsided. He died on the 8th day of the operation from hectic fever.

## Figures and Tables

**Figure f1:**